# Association between social and family support and antenatal depression: a hospital-based study in Chengdu, China

**DOI:** 10.1186/s12884-019-2510-5

**Published:** 2019-11-19

**Authors:** Ying Hu, Ying Wang, Shu Wen, Xiujing Guo, Liangzhi Xu, Baohong Chen, Pengfan Chen, Xiaoxia Xu, Yuqiong Wang

**Affiliations:** 1 Department of Obstetrics and Gynecology, West China Second University Hospital, Chengdu, China; 2 Key Laboratory of Birth Defects and Related Diseases of Women and Children (Sichuan University), Ministry of Education, Sichuan University, Chengdu, China; 3 Reproductive Endocrinology and Regulation Laboratory, West China Second University Hospital, Sichuan University, Chengdu, 610041 China; 4 Department of Nursing, Chengdu Women’s and Children’s Central Hospital, School of Medicine, University of Electronic Science and Technology of China, Chengdu, China; 5 West China School of Medicine, Sichuan University, Chengdu, China

**Keywords:** Antenatal depression, Persistent depression, Risk factors, EPDS, China

## Abstract

**Background:**

Antenatal depression (AD) is considered as one of the major health burdens and has adverse effects on the outcome of expectant mothers and newborns. The present study aims to investigate the prevalence of antenatal depression (AD), and to explore the potential risk factors of AD among pregnant women in Chengdu, including personal background, related social factors, family factors and cognitive factors.

**Methods:**

The prospective nested case-control study included pregnant women who were in their second pregnancy and attended prenatal care at three tertiary hospitals and one regional hospital in Chengdu, China, between March 2015 and May 2016. Self-designed questionnaires were given to participants in their second and third trimesters to collect information on clinical and demographic characteristics, and a modified edition of Edinburgh Postnatal Depression Scale (EPDS) were used to measure AD. The logistic regression was applicated in analyses.

**Results:**

A total of 996 pregnant women were included in analysis. Ninety-three women suffered from AD symptoms only in their second trimester, 96 only in their third trimester, and 107 displayed persistent depression in both trimesters. In the univariate analyses, age and marital relationships were linked with AD occurrence in both second and third trimester. In addition, increasing age, full-time job, higher education level, and no gender preference of spouse were associated with reduced persistent depression. Multivariate analysis showed that gender preference and marital relationship were the potential risk factors of persistent depression.

**Conclusions:**

Age, marital relationship relationships, with parents-in-law, the negative recognition of this pregnancy and husband’s gender preference were found as risk factors of AD occurrence in some specific trimester. Gender preference of husbands and marital relationships were independently associated with persistent depression. These findings suggest that stronger family support can help improve mental health of pregnant women.

## Background

Antenatal depression (AD) is a major depressive disorder during pregnancy characterized by depressive symptoms including sadness or low mood, despondency, sleep disturbance, changes in appetite, suicidal ideation, feelings of worthlessness, loss of interest or pleasure, etc., which may lead to devastating sequelae for the expectant mothers and families [1]. According to a systematic review, the pooled prevalence of prenatal depression estimates 9.2% in high-income countries and 19.2% in low-income and middle-income countries [2]. Based on publications that included patients at different trimesters and with varying demo-socio-economic status and used various study methodologies, the prevalence rate of AD among Chinese pregnant women ranges from 4 to 46.11% [3–5]. In addition, the reported prevalence of AD may be underestimated on account of the lower rate of identification and treatment in pregnant women compared with non-pregnant women [1]. There is growing evidence that AD not only affects pregnancy and neonatal outcomes, but also leads to the postnatal depression [6]. In addition, AD has influence on offspring’s cognitive development, emotions and behaviors in childhood [7]. Much attention devoted to women with AD and efficacious treatment interventions are the keys of prenatal care. However, only about 50% AD patients are treated adequately at present [8]. What makes the treatment of AD more complicated is that many antidepressants cannot be simply applied due to potential side effects on the fetus [8].

AD may occur in any trimester during pregnancy. Persistent AD refers to depression that lasts for at least two trimesters among pregnant women. Multiple studies conducted in non-Asian populations showed that long-term or severe depression might change the hormone level in the body and reduce the uterine blood supply, thus increasing the risk of fatal distress, premature rupture of membranes and other complications [9]. However, few studies have explored the persistent AD in China.

Several factors have been found to be associated with an increased risk of AD. Based on literature, potential risk factors mainly included four aspects: personal background, social and family aspect, obstetric aspect and cognitive aspect. Psychological and psychiatric factors such as previous history of mental illness, socio-demographic and economic factors such as ethnicity, employment, housing condition and income, social support, family relationships and life events had effect on AD occurrence to a certain extent [10, 11]. In addition, other medical conditions regarding vomiting, anemia, and gestational diabetes might also be related to the development of AD [9]. Compared to western women, Chinese women seem more family-oriented and thus are more likely to be affected by family relationships. Meanwhile, Chinese society has strong desire to optimize the mental health of women and offspring [12]. Lau et al. [13] revealed financial support, marital status, family interpersonal relationships, and social support might contribute to AD patients living in the city of Chengdu, China, although they did not investigate the AD prevalence regarding a specific trimester.

We therefore performed a prospective nested case-control study to investigate the prevalence of AD among pregnant women in Chengdu, and to explore potential risk factors of AD occurrence and persistent depression in the pregnancy.

## Methods

This study was conducted between March 2015 and May 2016 in three tertiary hospitals and one regional hospital in Chengdu, one of the central cities in Southwest China. Questionnaires were given to pregnant women who were scheduled for antenatal screening in one of the four hospitals, and each woman completed the questionnaires twice (in their second trimester and third trimester of the pregnancy, respectively). The questionnaires included two parts: I. General information (self-designed questions): (a) demographic factors; (b) social support factors; (c) obstetric factors; (d) cognitive aspects; II. Measurement of AD: the modified edition of 10-item Edinburgh Postnatal Depression Scale (EPDS) by Wang et al [14, 15], which is more suitable for Chinese population and is widely used for the maternal depression screening in mainland China. In the Mainland Chinese version of EPDS, a cutoff of 9.5 was recommended when screening for clinic depression, with a sensitivity of 80.00% and specificity of 83.03%, respectively [15]. Participants were included if they: 1) were able to read and write Chinese and could complete the questionnaires; 2) agreed to participate in the survey; 3) had no existing psychological problems, such as depression, anxiety; 4) had no medical history of mental illness other than depression. This is based on information from the survey and related medical charts. A total of 1065 women meeting the above criteria were recruited. Among them, 55 women dropped out and the rest of 1010 women filled out two questionnaires. An additional 14 women were excluded from analysis due to “20% blank” in the questionnaires or failure of completing questionnaire at both trimesters, leaving 996 women with valid questionnaires in the final analysis.

Categorical data were described using frequencies, and continuous data were described using means and standard deviations (SD). A multivariate logistic regression analysis was employed to identify independent risk factors of antenatal depression in terms of socio-demographic aspect, cognitive aspect and family aspect. Variables were entered for multivariate logistic regression analysis if the *P* < 0.05 were considered statistically significant in the univariate analysis or if the variables were deemed clinically important regardless of the statistical significance. Estimated associations were described using odds ratios (OR) with 95% confidence intervals (CIs). SPSS 25.0 (SPSS Chicago, IL, USA) was used for all statistical analyses. *P* < 0.05 was considered statistically significant.

## Results

**Fig. 1:**
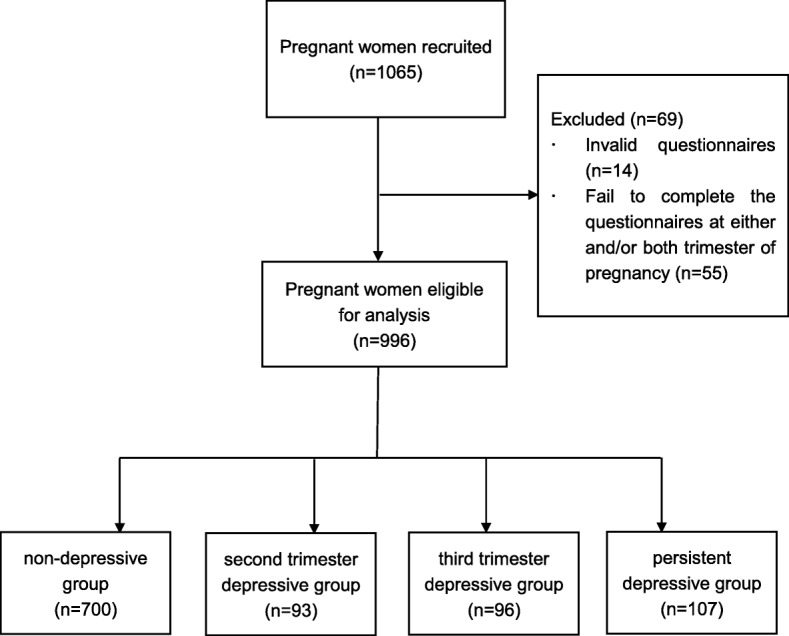
Flow diagram of the study

A total of 996 eligible pregnant women who completed questionnaires both in their second and third trimesters were included for analyses. They were divided into four categories: non-depressive group (no depression in neither of the two trimesters) (*n* = 700), second trimester-only depressive group (*n* = 93), third trimester-only depressive group (*n* = 96), and persistent depressive group (i.e., depressive at both time points, *n* = 107). The flow chart is demonstrated in Fig. 1.

Table 1 contains the socio-demographic characteristics, cognitive condition about this pregnancy, family background and obstetric characteristics of study participants. The mean age of the respondents were 28.93 years (SD =3.76). Results showed that most participants had lived in Chengdu for more than 5 years (*n* = 726, 72.89%), and the majority pregnant women had full-time job (*n* = 769, 77.21%) and higher education (*n* = 749, 75.20%). The housing area of few respondents (9.94%) spanned over 140 m^2^ and participants who owned housing property rights accounted for 86.45%.

**Table 1:** Baseline characteristics of included pregnant women

	Non-depressive group (*N* = 700, 70.28%)	Second trimester depressive group (*N* = 93, 9.34%)	Third trimester depressive group (*N* = 96, 9.64%)	persistent depressive group (*N* = 107, 10.72%)	Overall number (*N* = 996)
*Demographic data and social satus*					
*Maternal age (Mean ± SD)*	29.20 ± 3.80	28.32 ± 3.59	28.15 ± 3.67	28.40 ± 3.50	28.93 ± 3.76
*Educational level*					
Primary school	5 (0.71)	0 (0.00)	2 (2.08)	2 (1.87)	9 (0.90)
Secondary school	124 (17.71)	16 (17.20)	18 (18.75)	26 (24.30)	184 (18.47)
Bachelor and above	540 (77.14)	69 (74.19)	70 (72.92)	70 (65.42)	749 (75.20)
Unknown	31 (4.43)	8 (8.60)	6 (6.25)	9 (8.41)	54 (5.42)
*Employment*^a^					
Part-time job	24 (3.43)	4 (4.30)	4 (4.17)	1 (0.93)	33 (3.31)
Full time job	546 (78.00)	72 (77.42)	75 (78.13)	76 (71.03)	769 (77.21)
Unemployed	130 (18.57)	17 (18.28)	17 (17.71)	30 (28.04)	194 (19.48)
*Years of living in Chengdu*					
< 5 years	183 (26.14)	28 (30.11)	27 (28.13)	32 (29.91)	270 (27.11)
6–10 years	196 (28.00)	27 (29.03)	33 (34.38)	33 (30.84)	289 (29.02)
11–20 years	105 (15.00)	13 (13.98)	6 (6.25)	14 (13.08)	138 (13.86)
> 20 years	216 (30.86)	25 (26.88)	30 (31.25)	28 (26.17)	299 (30.02)
*Family income* per capita *(Yuan)*					
< 1200	21 (3.00)	3 (3.23)	2 (2.08)	3 (2.80)	29 (2.91)
1200–2000	69 (9.86)	6 (6.45)	12 (12.50)	13 (12.15)	100 (10.04)
2001–3000	101 (14.43)	15 (16.13)	20 (20.83)	20 (18.69)	156 (15.66)
3001–4000	141 (20.14)	19 (20.43)	15 (15.63)	19 (17.76)	194 (19.48)
> 4000	368 (52.57)	50 (53.76)	47 (48.96)	52 (48.60)	517 (51.91)
*Types of residence*					
Have property rights	606 (86.57)	79 (84.95)	84 (87.50)	92 (85.98)	861 (86.45)
No property rights	94 (13.43)	14 (15.05)	12 (12.50)	15 (14.02)	135 (13.55)
*Size of residence*					
< 90 m^2^	315 (45.00)	46 (49.46)	46 (47.92)	53 (49.53)	460 (46.18)
90–140 m^2^	309 (44.14)	40 (43.01)	43 (44.79)	45 (42.06)	392 (39.36)
> 140 m^2^	76 (10.86)	7 (7.53)	7 (7.29)	9 (8.41)	99 (9.94)
*Cognitive factors*					
*Degree of pregnancy knowledge*					
Incomprehension	4 (0.57)	0 (0.00)	0 (0.00)	1 (0.93)	5 (0.50)
General understanding	662 (94.57)	90 (96.77)	91 (94.79)	101 (94.39)	944 (94.78)
Deep understanding	34 (4.86)	3 (3.23)	5 (5.21)	5 (4.67)	47 (4.72)
*Attending maternity school*					
Yes	388 (55.43)	47 (50.54)	57 (59.38)	70 (65.42)	562 (56.43)
No	312 (44.57)	46 (49.46)	39 (40.63)	37 (34.58)	434 (43.57)
*Planned pregnancy*					
Intended pregnancy	529 (75.58)	63 (67.74)	73 (76.04)	73 (68.22)	738 (74.25)
Unintended pregnancy	158 (22.64)	26 (27.96)	22 (22.92)	32 (29.91)	238 (23.94)
Unknown	13 (1.58)	4 (4.30)	1 (1.04)	2 (1.87)	20 (2.01)
*The recognition of this pregnancy*					
Task	44 (6.29)	6 (6.45)	15 (15.63)	10 (9.35)	75 (7.53)
Burden	26 (3.71)	4 (4.30)	7 (7.29)	5 (4.67)	42 (4.22)
Pleasure	625 (89.29)	83 (89.25)	74 (77.08)	89 (83.18)	871 (87.45)
Other	5 (0.71)	0 (0.00)	0	3 (2.80)	8 (0.80)
*Concern for fetal health*					
Yes	651 (93.13)	87 (93.55)	92 (95.83)	103 (96.26)	933 (93.77)
No	48 (6.87)	6 (6.45)	4 (4.17)	4 (3.74)	62 (6.23)
*Family support*					
*Gender expectations for the fetus of the spouse*					
No	575 (82.14)	75 (80.65)	70 (72.92)	76 (71.03)	796 (79.92)
Boys	59 (8.43)	9 (9.68)	14 (14.58)	12 (11.21)	94 (9.44)
Girls	66 (9.43)	9 (9.68)	12 (12.50)	19 (17.76)	106 (10.64)
*Marital relationship in the last 3 months*^a^					
Good	649 (92.71)	72 (77.42)	84 (87.50)	78 (72.90)	883 (88.65)
General	50 (7.14)	21 (22.58)	12 (12.50)	27 (25.23)	110 (11.04)
Poor	2 (1.87)	0 (0.00)	0 (0.00)	1 (0.14)	3 (0.30)
*Relationship with parents-in-law in the last 3 months*					
Good	594 (84.86)	80 (86.02)	75 (78.13)	94 (87.85)	843 (84.64)
General	104 (14.86)	12 (12.90)	19 (19.79)	12 (11.21)	147 (14.76)
Poor	2 (0.29)	1 (1.08)	2 (2.08)	1 (0.93)	6 (0.60)
*Relationship with own parents in the last 3 months*					
Good	658 (94.00)	92 (98.92)	87 (90.63)	103 (96.26)	940 (94.38)
General	42 (6.00)	1 (1.08)	9 (9.38)	4 (3.74)	56 (5.62)
*Obstetric factors*					
*Gravidity (Mean ± SD)*	2.09 ± 1.27	2.29 ± 1.54	1.96 ± 129	2.23 ± 1.43	2.11 ± 1.31
*Parity (Mean ± SD)*	0.08 ± 0.30	0.03 ± 0.18	0.09 ± 0.33	0.09 ± 0.29	0.08 ± 0.29
*Spontaneous abortion (Mean ± SD)*	0.13 ± 0.43	0.26 ± 0.66	0.16 ± 0.49	0.27 ± 0.70	0.16 ± 0.50
*Induced abortion (Mean ± SD)*	0.90 ± 1.12	0.99 ± 1.33	0.80 ± 0.97	0.99 ± 1.20	0.91 ± 1.14
*Morning sickness*					
Yes	597 (85.29)	81 (87.10)	87 (90.625)	99 (92.52)	864 (86.75)
No	103 (14.71)	12 (12.90)	9 (9.375)	8 (7.48)	132 (13.25)
*Previous history about obstetrical and gynecological disease*					
Yes	406 (0.58)	5356.99)	61 (63.54)	72 (67.29)	592 (59.44)
No	294 (0.42)	40 (43.01)	35 (36.46)	35 (32.71)	404 (40.56)
*Assisted reproduction*					
Yes	12 (1.71)	2 (2.15)	0 (0.00)	3 (2.80)	17((1.71)
No	688 (98.29)	91 (97.85)	96 (100.00)	104 (97.20)	979 (98.29)

*N* number of included pregnant women *m*^*2*^ square meters; *SD* standard deviation

^a^ Missing data

As for the cognitive aspect, majority of the pregnant women had a general understanding of pregnancy knowledge (*n* = 944, 94.78%) and had planned for this pregnancy (*n* = 738, 74.25%). In addition, 87.45% of them were happy with the pregnancy and 93.77% concerned for the fetal health.

Displayed in the family aspect was, around 20% of participants (*n* = 200) reported that their spouses had gender preference. There were 88.65% participants having good relationship with their husbands in the last 3 months, 843 (84.64%) participants had close relationship with their parents-in-law and 94.38% had good relationship with their own parents. Considering the obstetric characteristics, the mean number of times of gravidity, parity, spontaneous abortion, induced abortion was 2.11, 0.08, 0.16 and 0.91, respectively. 59.44% pregnancy women had history of gynecological and obstetric diseases such as dysmenorrhea and dystocia.

Table 2 contains the results of univariate of social support. Compared with non-depressive group, older age was a protective factor for the occurrence of antenatal depression (second trimester depressive group vs. non-depressive group: (OR = 0.938, 95%CI: 0.883–0.996, *P* < 0.05); third trimester depressive group vs. non-depressive group: (OR = 0.925, 95%CI: 0.871–0.982, *P* < 0.05); persistent depressive group vs. non-depressive group: (OR = 0.944, 95%CI: 0.892–0.998, *P* < 0.05)). Similarly, participants with full-time job (OR = 0.603, 95%CI: 0.379–0.959, *P* < 0.05) and higher education (OR = 0.608, 95%CI: 0.393–0.940, *P* < 0.05) in the persistent depressive group also showed lower likelihood of depression.

**Table 2:** Univariate analysis of the association between sociodemographic characteristics and antenatal depression

	Second trimester depressive group (*N* = 93)	Third trimester depressive group (*N* = 96)	persistent depressive group (*N* = 107)
	OR (95% CI)	OR (95% CI)	OR (95% CI)
*Age*	0.938 (0.883,0.996) ^b^	0.925 (0.871,0.982) ^b^	0.944 (0.892,0.998) ^b^
*Educational level*	1.073 (0.619,1.860)	0.795 (0.489,1.294)	0.608 (0.393,0.940) ^b^
*Family income* per capita	1.045 (0.861,1.268)	0.913 (0.764,1.091)	0.915 (0.772,1.085)
*Size of residence*	0.832 (0.593,1.166)	0.859 (0.617,1.195)	0.848 (0.618,1.164)
*Years of living in Chengdu*			
≤ 5 years	1.322 (0.745,2.347)	1.062 (0.609,1.852)	1.349 (0.783,2.324)
6–10 years	1.190 (0.668,2.120)	1.212 (0.713,2.061)	1.299 (0.757,2.228)
11–20 years	1.070 (0.526,2.175)	0.411 (0.166,1.019)	1.029 (0.520,2.036)
> 20 years ^a^			
*Employment*			
Part-time	1.275 (0.394,4.119)	1.275 (0.394,4.119)	0.181 (0.023,1.388)
Full time	1.008 (0.575,1.769)	1.050 (0.600,1.839)	0.603 (0.379,0.959) ^b^
Unemployed ^a^			
*Types of residence*			
Have property rights	0.875 (0.476,1.608)	1.086 (0.571,2.065)	0.951 (0.529,1.712)
No property rights ^a^			

*N* number of included pregnant women; *OR* odds ratio; *CI* confidence interval

^a^ Reference

^b^ Statistically significant

Regarding family support and cognitive aspect, AD was significantly lower in pregnant women whose husbands had no gender preference (OR = 0.459, 95%CI: 0.261–0.807, *P* < 0.05). Poor marital relationship was a risk factor for second trimester depression (OR = 3.419, 95%CI: .980–5.904, *P* < 0.05) and third trimester depression (OR = 4.624, 95%CI: 2.830–7.555, *P* < 0.05). Poor relationships with parents-in-law or own parents were significant predictors of the third trimester depression (OR = 1.635, 95%CI: 1.010–2.646, *P* < 0.05) in third trimester depressive group. Pregnant women who considered the pregnancy as a task also had elevated risk of depression in the third trimester (OR = 2.879, 95%CI: 1.528–5.426, *P* < 0.05). (Table 3).

**Table 3:** Univariate analysis of risk factors associated with antenatal depression in family support and cognition aspects among pregnant women

	Second trimester depressive group (*N* = 93)	Third trimester depressive group (*N* = 96)	persistent depressive group (*N* = 107)
	OR (95% CI)	OR (95% CI)	OR (95% CI)
*Degree of knowledge of pregnancy*	0.798 (0.286,2.232)	1.186 (0.484,2.906)	0.894 (0.351,2.274)
*Relationship with husband in the last 3 months*	3.419 (1.980,5.904) ^b^	4.624 (2.830,7.555) ^b^	1.748 (0.908,3.366)
*Relationship with parents-in-law in the last 3 months*	0.974 (0.545,1.741)	1.635 (1.010,2.646) ^b^	0.836 (0.469,1.491)
*Relationship with own parents in the last 3 months*	0.170 (0.023,1.252)	1.621 (0.763,3.444)	0.608 (0.214,1.732)
*Attending maternity knowledge lecture*			
Yes	1.217 (0.789,1.877)	0.851 (0.551,1.313)	0.657 (0.430,1.006)
No ^a^			
*Planned pregnancy*			
Intended pregnancy	0.724 (0.443,1.182)	0.991 (0.596,1.648)	0.681 (0.434,1.071)
Unintended pregnancy ^a^			
*The recognition of this pregnancy*			
Task	1.027 (0.425,2.483)	2.879 (1.528,5.426) ^b^	1.596 (0.776,3.284)
Burden	1.158 (0.394,3.402)	2.274 (0.954,5.420)	1.350 (0.506,3.607)
Pleasure ^a^			
*Concern for fetal health*			
Yes	1.069 (0.444,2.572)	1.696 (0.598,4.813)	1.899 (0.670,5.377)
No ^a^			
*Spouse gender expectations for the fetus*			
No	0.957 (0.458,1.998)	0.670 (0.345,1.300)	0.459 (0.261,0.807) ^b^
Girls	1.119 (0.416,3.006)	1.305 (0.559,3.045)	0.707 (0.316,1.578)
Boys ^a^			

*N* number of included pregnant women; *OR* odds ratio; *CI* confidence interval

^a^ Reference

^b^ Statistically significant

As showed in Table 4, seven factors entered the multivariate logistic regression analysis. According to the result, maternal age was independent risk factor both in second trimester depressive group and third depressive group. Poor relationship with parents-in-law and pregnant women who considered pregnancy as task were associated with higher third trimester depression. Additionally, poor marital relationship with the last 3 months was a predictor factor for pregnant women in second trimester depressive group (OR = 3.188, 95%CI: 1.759–5.780, *P* < 0.05) and persistent depressive group (OR = 4.772, 95%CI: 2.792–8.155, *P* < 0.05). Furthermore, no spouse gender expectation for the fetus was a protective factor of persistent depression of pregnant women (OR = 0.400, 95%CI: 0.221–0.726, *P* < 0.05).

**Table 4:** Multivariate logistic regression analysis of potential risk factors of antenatal depression

	Second trimester depressive group (*N* = 93)	Third trimester depressive group (*N* = 96)	persistent depressive group (*N* = 107)
	OR (95% CI)	OR (95% CI)	OR (95% CI)
*Age*	0.928 (0.868,0.991) ^b^	0.918 (0.861,0.980) ^b^	0.951 (0.895,1.011)
*Educational level*	1.293 (0.713,2.344)	0.947 (0.562,1.597)	0.775 (0.470,1.277)
*Relationship with husband in the last 3 months*	3.188 (1.759,5.780) ^b^	1.571 (0.793,3.115)	4.772 (2.792,8.155) ^b^
*Relationship with parents-in-law in the last 3 months*	0.870 (0.452,1.674)	1.796 (1.070,3.017) ^b^	0.765 (0.397,1.475)
*Employment*			
Part-time job	1.075 (0.283,4.075)	1.401 (0.415,4.722)	–
Full time job	1.026 (0.554,1.899)	0.932 (0.510,1.703)	0.918 (0.524,1.611)
Unemployed ^a^			
*The recognition of this pregnancy*			
Task	0.947 (0.373,2.404)	2.850 (1.424,5.702) ^b^	0.865 (0.360,2.080)
Burden	1.323 (0.430,4.069)	2.316 (0.925,5.802)	0.993 (0.321,3.074)
Pleasure ^a^			
*Gender expectations for the fetus of the spouse*			
No	1.034 (0.471,2.267)	0.627 (0.318,1.239)	0.400 (0.221,0.726) ^b^
Girls	1.028 (0.341,3.094)	1.164 (0.478,2.831)	0.702 (0.294,1.673)
Boys ^a^			

*N* number of included pregnant women; *OR* odds ratio; *CI* confidence interval

^a^ Reference

^b^ Statistically significant

## Discussion

To our knowledge, this is the first study investigating persistent AD across the second and third trimesters of pregnant women without interventions in Southwest China. Results of univariate analyses showed that older age, full-time job and higher educational levels were negatively associated with risk of persistent AD in their second and third trimesters. Regarding the family support, spouses of the participants with no gender preference may favorably affect the outcome of persistent depression. Moreover, the relationships with husbands and with parents-in-law had disproportionate influence on the antenatal depression of the pregnant women in their second or third trimester. In the multivariate analysis, pregnant women whose husbands had no expectations of fetal sex or got on well with them were less likely to get overwhelmed by persistent AD. However, women exposed to detrimental relationships with their parents-in-law had a significant elevated risk of AD in the third trimester. The results showed that the mutual influence of social support and family support might play a crucial role in antenatal depression of pregnant women in different trimesters in Chengdu.

Recently, there is evidence suggesting that AD is becoming increasingly prevalent. Therefore, studies on specific groups who were at high risk of developing depression may assist in early identification, intervention and prevention [11, 16]. *Gavin* et al. [17] reported a decrease in the AD prevalence rates as delivery period drawing near, whereas *Bennett* et al [18] found rates in the second and third trimesters almost as twice as the first trimester. Despite the limited local evidence and large disparity of these studies, the results indicated that high rate of AD tended to occur in women who lived in the context of social and family adversity. Compared with Western women, Chinese women tended to implicitly manifest their depression through somatic complaints thus the reported incidence were possibly underestimated in the present study [19]. Besides, crude findings in Bayrampour et al [20] similarly found 11.1% pregnant women admitted in the study, and the study showed that the prevalence of persistent depression lasted in the second-third trimester group was 2.5%, which was lower than transient depression (symptomatic exclusively in the second trimester) [20]. However, the duration of depressive status may be linked with the severity of adverse maternal and child outcomes, which may be attributed to the alterations in hyperactive hypothalamo-pituitary-adrenal cortical axis and the inflammatory system [21]. Our results extended the risk factors in transient AD to the common AD risk factors persisting in second and third trimesters, aiding in the integration of trajectories of perinatal depression and the development of the underlying mechanisms and causal pathways in which poor depressive status leads to adverse outcomes.

The results of both univariate and multivariate analysis revealed that maternal age was an important sociodemographic risk factor in AD, which had been demonstrated in several studies [10, 13]. Previous studies showed that younger women had an increased risk of AD due to the vulnerability and dependency when they were young [13, 22, 23]. However, one study [5] conducted in Shanghai has opposite finding, showing that older women were more likely to have AD because more pregnant complications and stressful life events were associated with the higher AD rates in older pregnant women. The divergence might be related to the different age ranges of pregnant women included in the studies [24]. For example, pregnant women with older maternal age might have more complications, which might be regarded as “confounding by indication” [24]. This was also the potential explanation that older maternal age was not considered as an independent predictor for second and third persistent depressive group in multivariate model. Consistent with previous studies, employment and higher educational level of pregnant women were related to the AD prevalence [25]. It has been found that women in the setting of full-time work and higher educational level had good social support, and they were less likely to have economic concern and to have pessimistic view on pregnancy complications. Instead, they were more likely to seek medical help for any pain or stress relief [18, 26]. However, the two factors were not statistically significant in multivariate analysis. One possible reason was that senior intellectual females might encounter with more difficulties and challenges with the expansion of high education and increased female employment in China, and they were also more likely to experience the rapid shift in family values. Therefore, they may postpone childbearing to high-risk age for pregnancy [27]. Another possible explanation was that individuals with good education were more sensitive to and not embarrassed about admitting depression [28]. Our finding did not show any association between years of living in Chengdu and AD incidence, which was not consistent with the findings from Lau et al. [13]. Lau et al. highlighted the importance of migrant-related factors occurring in the rapidly developing country [13]. However, with the rapid development of Chengdu, the living environment and public welfare for new migrants and members of their families, such as subsidized housing, education, and medical benefits, have been dramatically improved so that women with shorter time in Chengdu could be free from these issues.

Of the family-related factors, relationships with family members, especially with husband, had valuable prognostic significance for AD. Although it has been well known that active support from partner was a pivotal instrument in the mental health of pregnant women, support from extended family was also of importance for the consanguinity relationship and the idea of conformity in Chinese culture [12]. Likewise, the traditional preference for boys to girls are still deeply rooted in Chinese people, especially in rural areas. *Adewuya* et al. [29] found the delivery of a female baby was a predictor of postnatal depression, which was consistent to our finding to some extent. As the result showed, spouse gender expectations for the baby and the relationship with husband were statistically associated with second-third trimester AD, suggesting that practical support, such as no gender preference for fetus, might reduce the undesirable psychological burden of pregnant women [11].

Apart from the well-known risk factors, a longitudinal pregnancy cohort with the similar setting, summarized three elements as the risk factors of chronic depressive and anxiety symptoms, including high perceived stress, low social support, and the history of mental health problems, which were consistent with our results to some extent [20]. *Mora* et al. showed multiparous women were more likely to be chronically depressive compared with other women [30] and *Chung* et al. noted that persistent depressive symptoms through the perinatal period was related to poor parenting practices [31]. Additionally, biological variables did act on AD indirectly through their significant effects on psychosocial stressors and symptoms of perinatal mood disorders [32].

There were some limitations of this study. Firstly, the study was hospital-based, and the representative of the sample was limited if pregnant women with AD were unwilling or unable to attend the screening. Meanwhile, patients with some physical or mental illness which were also considered as risk factors of AD are more likely to be excluded from the study. Secondly, data collected from the self-report psychometric questionnaires may be subject to recall bias. Thirdly, we did not evaluate the obstetrical risk factors in the present study, and we were unable to determine whether it is causal association between AD and risk factors mentioned above. Besides, too many obstetrical factors need to be taken into account and some factors such as pregnancy complications are changeable along with the pregnant period. Moreover, these limitations will hopefully be addressed in future research.

## Conclusions

In conclusion, the current mental health of pregnant women during pregnant is not optimistic in Chengdu. Of these AD women, more than a third of pregnant women represent persistent depressive symptoms. In addition, there are some factors related to AD in specific trimester or in persistent depression among pregnant women in Chengdu, including maternal age, the recognition of the pregnancy, spouse sex expectations for the fetus, and relationships with family members. The study may help professionals identify high-risk pregnant women and help them collaborate with family members to improve the mental health of these women. Further research on exposure factors and the effective interventions of AD is warranted.

## Data Availability

The datasets used and analyzed during the current study are available from the corresponding author on reasonable request.

## Abbreviations

AD: Antenatal depression
EPDS: Edinburgh Postnatal Depression Scale
m^2^: Square meters
OR: Odds ratios
RMB: Renminbi
SD: Standard deviations
